# Spatial working memory performance in people with obsessive–compulsive disorder, their unaffected first-degree relatives and healthy controls

**DOI:** 10.1192/bjo.2021.1052

**Published:** 2021-11-15

**Authors:** Stephan Heinzel, Katharina Bey, Rosa Grützmann, Julia Klawohn, Christian Kaufmann, Leonhard Lennertz, Michael Wagner, Norbert Kathmann, Anja Riesel

**Affiliations:** Department of Psychology, Humboldt Universität zu Berlin, Germany; and Department of Education and Psychology, Freie Universität Berlin, Germany; Department of Psychiatry and Psychotherapy, University Hospital Bonn, Germany; and German Center for Neurodegenerative Diseases (DZNE), Germany; Department of Psychology, Humboldt Universität zu Berlin, Germany; Department of Psychology, Humboldt Universität zu Berlin, Germany; Department of Psychology, Humboldt Universität zu Berlin, Germany; Department of Psychiatry and Psychotherapy, University Hospital Bonn, Germany; Department of Psychiatry and Psychotherapy, University Hospital Bonn, Germany; and German Center for Neurodegenerative Diseases (DZNE), Germany; and Department for Neurodegenerative Diseases and Geriatric Psychiatry, University Hospital Bonn, Germany; Department of Psychology, Humboldt Universität zu Berlin, Germany; Department of Psychology, Humboldt Universität zu Berlin, Germany; and Department of Psychology, University of Hamburg, Germany

**Keywords:** Obsessive–compulsive disorder, spatial working memory, endophenotype, first-degree relatives, risk assessment

## Abstract

Studies have shown that people with obsessive–compulsive disorder (OCD) have impairments in spatial working memory (SWM) performance. However, it remains unclear whether this deficit represents a cognitive endophenotype preceding symptoms or a correlate of OCD. We investigated SWM in 69 people with OCD, 77 unaffected first-degree relatives of people with OCD and 106 healthy control participants. Taking age effects into account, SWM performance was best in healthy controls, intermediate in relatives and worst in OCD participants. However, since performance did not differ significantly between healthy controls and relatives, our study does not fully support SWM performance as a core cognitive endophenotype of OCD.

Obsessive–compulsive disorder (OCD) is a relatively common mental disorder (2–3% of the population) that is characterised by intrusive and unwanted obsessive thoughts and compulsive behaviours. Increasing evidence from neuropsychological and neuroimaging studies suggests that dysfunctions in frontoparietal and frontostriatal brain circuitry in OCD^1,2^ related to performance deficits in executive control tasks^3^ are key psychopathological mechanisms. Studies in unaffected first-degree relatives of people with OCD have been conducted to examine whether specific neurobiological and cognitive alterations may serve as endophenotypes or risk markers for OCD.^4–6^ However, to date, it remains uncertain which specific domain within the range of executive functions fulfils criteria for a candidate endophenotype. As yet, impaired performance on spatial working memory (SWM) tasks has been reported repeatedly in people with OCD and linked to obsessive–compulsive symptoms.^3^ However, there is little research on this cognitive domain in unaffected relatives of people with OCD,^5,7^ and more research is needed to decide whether SWM performance may be a core candidate for endophenotypes of OCD. The current study in 252 people with OCD, their unaffected first-degree relatives and healthy control participants is the largest of its kind and seeks to contribute to this initiative. It is hypothesised that both the OCD participants and the relatives would perform worse on an SWM task compared with healthy controls.

## Method

### Participants

Sixty-nine people with OCD, 77 relatives and 106 healthy controls without a family history of OCD participated in the study. Past and present mental disorders were assessed in all participants using the German version of the Structured Clinical Interview for DSM-IV TR Axis I Disorders: Research Version, Patient Edition (SCID-I/P). Information on psychopathology of the first-degree relatives of all participants was obtained by the Family History Screen to ensure that healthy controls were free of a family history of OCD. The study was approved by the local Ethics Committees of the Humboldt-Universitaet zu Berlin and the University Hospital Bonn, and written informed consent was obtained from all participants. See the supplementary material available at https://doi.org/10.1192/bjo.2021.1052 for further information on sample characteristics, exclusion criteria and additional references.

### Procedure

Please refer to the Supplementary Methods and the Supplementary Fig. 1 for more details on the task set-up. The goal of the task was to find blue tokens hidden in boxes. Participants were told that once a token was found in a particular box, the next tokens were hidden in different boxes. We employed a moderately difficult version of the SWM test, with 11 problems, from the Cambridge Neuropsychological Test Automated Battery. Outcome measures of SWM performance were between-search errors (sum of trials where a participant returned to a box in which a token had already been found) and strategy scores (sum of search sequences that started from a novel box for 6-, 8- and 10-box problems). High strategy scores indicate an inability to adopt a systematic search strategy (i.e. starting each search sequence from the same box until a token is found in that box^8^). Strategy scores and between-search errors are both indicators of SMW performance and were highly correlated (*r* = 0.58, *P* < 0.001). Thus, to obtain a robust measure of SWM performance, the two parameters were combined by computing the mean of the *z*-transformed variables for further analyses. Note that higher values indicate worse SWM performance. See Supplementary Tables 1 and 2 for all raw scores and additional analyses using the individual variables.

Statistical analyses were conducted using the Statistical Package for the Social Sciences, Release 27.0 for Windows. To investigate differences in SWM performance between OCD participants, relatives and healthy controls, we conducted an analysis of covariance (ANCOVA) using group as the factor, age as the covariate and SWM performance as the dependent variable. Age was included as the covariate since relatives were older than OCD participants (*t*(144) = 7.73, *P* < 0.001) and healthy controls (*t*(181) = 8.24, *P* < 0.001), and age was related to SWM performance (*r* = 0.37, *P* < 0.001) in the entire sample. To verify results from the ANCOVA, we built a random age-matched subsample and conducted an ANOVA without age covariation as a sensitivity analysis. Case–control matchings were performed using the MedCalc software version 19.6.4 for Windows (https://www.medcalc.org). Individuals in the three groups were matched for age with a maximum allowed difference of 3 years (100 iterations). The subsamples were drawn randomly from the whole sample, resulting in 34 OCD participants, 34 relatives and 34 healthy controls. We report the effect size partial η² for the ANCOVA and Cohen's *d* for *t*-tests.

## Results

The ANCOVA in the whole sample revealed a significant group effect (*F*(2, 248) = 4.45, *P* = 0.013, partial η² = 0.035). Taking the effect of age into account, SWM performance was estimated to be best in healthy controls, intermediate in relatives and worst in OCD participants (Table 1, whole sample results). *Post hoc* comparisons indicated a significant difference between healthy controls and OCD participants (*t*(173) = 2.88, *P* = 0.005, *d* = 0.45). However, SWM performance in relatives did not differ significantly from that in healthy controls (*t*(181) = 1.09, *P* = 0.279, *d* = 0.16) or OCD participants (*t*(144) = 1.60, *P* = 0.112, *d* = 0.27). In the age-matched subsample (Table 1), a very similar performance pattern was observed (*F*(2, 99) = 3.49, *P* = 0.034, partial η² = 0.066). Healthy controls showed better SWM performance compared with OCD participants (*t*(66) = 2.77, *P* = 0.007, *d* = 0.67) and SWM performance in relatives was intermediate between healthy controls and OCD participants, but did not differ significantly from healthy controls (*t*(66) = 1.38, *P* = 0.172, *d* = 0.34) or OCD participants (*t*(66) = 1.21, *P* = 0.232, *d* = 0.29) (Supplementary Fig. 2). There were no significant effects of medication status or depressive comorbidity on SWM performance in the whole sample or in the age-matched subsample (all *P* > 0.61).

**Table 1:** Participants’ demographics^a^

Measure	Whole sample (*n* = 252)				Age-matched subsample (*n* = 102)			
	Healthy controls (*n* = 106)	Relatives (*n* = 77)	OCD (*n* = 69)	*F* or χ², *P*	Healthy controls (*n* = 34)	Relatives (*n* = 34)	OCD (*n* = 34)	*F* or χ², *P*
Age, years: mean (s.d.)	33.69 (12.31)	49.00 (12.56)	33.20 (12.06)	*F* = 42.36, *P* < 0.001	40.50 (13.52)	40.59 (13.79)	39.94 (13.36)	*F* = 0.02, *P* = 0.997
Gender, male/female, *n*	43/63	23/54	34/35	χ² = 5.79, *P* = 0.055	12/22	8/26	17/17	χ² = 5.17, *P* = 0.075
Verbal IQ (WST score): mean (s.d.)	104.46 (10.21)	106.99 (10.53)	101.65 (10.83)	*F* = 4.72, *P* = 0.010	105.76 (11.06)	104.18 (9.60)	104.32 (12.41)	*F* = 0.21, *P* = 0.808
Y-BOCS severity scale score, mean (s.d.)			21.49 (7.35)				22.23 (7.96)	
OCI-R sum score, mean (s.d.)	4.36 (4.15)	7.14 (7.11)	29.90 (12.89)	*F* = 221.12, *P* < 0.001	4.32 (4.30)	6.56 (6.48)	28.26 (12.12)	*F* = 86.03, *P* < 0.001
BDI-2 sum score, mean (s.d.)	3.10 (3.84)	5.88 (7.11)	17.61 (10.18)	*F* = 92.66, *P* < 0.001	3.50 (3.82)	6.50 (7.61)	19.18 (10.22)	*F* = 39.88, *P* < 0.001
SMW performance (z-score),^b^ mean (s.d.)	−0.17 (0.83)	−0.03 (0.90)	0.20 (0.83)	*F* = 4.45, *P* = 0.013	−0.22 (0.81)	0.08 (0.97)	0.35 (0.89)	*F* = 3.49, *P* = 0.034

OCD, participants with obsessive–compulsive disorder; SWM, spatial working memory; WST, Wortschatztest (German-language verbal IQ test); Y-BOCS, Yale–Brown Obsessive Compulsive Scale; OCI-R, Obsessive–Compulsive Inventory – Revised; BDI-II, Beck Depression Inventory – II.

a. Note that 24 (14 in the age-matched subsample) participants with OCD had a comorbid depressive disorder and 38 (21 in the age-matched subsample) took psychotropic medication.

b. *z*-scores in the whole sample represent estimated marginal means from the ANCOVA model with the covariate age; *z*-scores in the age-matched subsample represent estimated marginal means from the ANOVA model. Higher values indicate worse SWM performance. See Supplementary Table 1 for all raw scores and Supplementary Table 2 for additional analyses with the individual variables strategy score and between-search errors.

## Discussion

In the present study, SWM performance as indicated by between-search errors and strategy scores was investigated in people with OCD, their unaffected relatives and healthy controls. We replicated an SWM impairment in OCD as reported in previous studies.^3^ Significant effects in both the whole sample and the age-matched subsample suggest that SWM performance is robustly impaired in OCD. Relatives showed an intermediate performance on the SWM task. Small effect sizes of SWM performance differences between relatives and healthy controls are comparable to those in previous studies.^5,7^ When also considering performance differences between the OCD participants and healthy controls, this pattern could be seen in line with a genetically determined endophenotype. However, since the critical contrasts between relatives and healthy controls were not significant, our study does not fully support the notion of SWM performance impairment as a core candidate for endophenotypes of OCD. Notably, with the largest study of its kind, we were able to control for age-related effects through both covariance and sensitivity analyses, which has not been done in most previous studies.

As indicated by neuroimaging studies, endophenotypes of OCD may be characterised on the level of neuronal circuits rather than the level of task performance. A study by de Vries and colleagues^4^ tested a spatial *n*-back working memory task and did not detect performance deficits in siblings of people with OCD but found compensatory neural activity in frontoparietal circuits. Consequently, performance deficits in relatives are suggested to become visible mainly in highly executively demanding tasks if neural capacity^9^ is exceeded, for example in planning tasks.^10^ Future studies in SWM could include more executively demanding task variants, apply neuroimaging during SWM performance and use even larger samples to explore moderating variables. A better understanding of the mechanisms of SWM dysfunctionality in the development and maintenance of OCD could help in designing prevention programmes for persons at high risk for OCD and improve OCD treatments, for example by adding specific cognitive training.

## Data Availability

Data ares available from the corresponding author on request.

## Supplementary material

For supplementary material accompanying this paper visit http://dx.doi.org/10.1192/bjo.2021.1052. click here to view supplementary material
