# Supplementary material for: Spatial working memory performance in people with obsessive–compulsive disorder, their unaffected first-degree relatives and healthy controls
Source: BJPsych Open. 2021 Nov 15;7(6):e208. doi: 10.1192/bjo.2021.1052 (PMC8612012; doi:10.1192/bjo.2021.1052)
Supplement: Supplementary file 1 [file S2056472421010528sup001.docx]

**SUPPLEMENTARY MATERIAL**

for

**Spatial working memory performance in patients with obsessive-compulsive disorder, their unaffected first-degree relatives, and healthy controls**

*Stephan Heinzel^1,2^ *, Katharina Bey^3,4^, Rosa Grützmann^1^, Julia Klawohn^1^, Christian Kaufmann^1^, Leonhard Lennertz^3^, Michael Wagner^3,4,5^, Norbert Kathmann^1^, & Anja Riesel^1,6^*

^1^ Department of Psychology, Humboldt Universität zu Berlin, Rudower Chaussee 18, 12489 Berlin, Germany;

^2^ Department of Education and Psychology, Freie Universität Berlin, Habelschwerdter Allee 45, 14195 Berlin, Germany;

^3^ Department of Psychiatry and Psychotherapy, University Hospital Bonn, Venusberg-Campus 1, 53127 Bonn, Germany;

^4^ German Center for Neurodegenerative Diseases (DZNE), Venusberg-Campus 1, 53127 Bonn, Germany;

^5^ Department for Neurodegenerative Diseases and Geriatric Psychiatry, University Hospital Bonn, Venusberg-Campus 1, 53127 Bonn, Germany;

^6^ Department of Psychology, University of Hamburg, Von-Melle-Park 11, 20146 Hamburg, Germany.

**Supplementary Methods**

**Supplementary information on sample characteristics**

Patients were excluded if they had a current or lifetime diagnosis of psychotic, bipolar, or substance use disorder, or if they took neuroleptic medication in the past four weeks or benzodiazepines in the past two weeks. First-degree relatives of patients with OCD were excluded if the SCID did not verify the diagnosis of their affected relative or if they had a current or lifetime diagnosis of OCD, psychotic, bipolar, or substance use disorder. Relatives were also excluded if they took any psychotropic medication in the past four weeks.

OCD patients and relatives were recruited via the outpatient clinics at the Department of Psychology of Humboldt Universitaet zu Berlin and at the Department of Psychiatry and Psychotherapy of the University Hospital Bonn, Germany. Healthy control participants were recruited via online and public advertisements and were matched for age, gender, and education level to the OCD patients. The following exclusion criteria applied to healthy controls: psychotropic medication in the past 3 months, any current or past mental disorder according to DSM-IV TR axis-I, family history of OCD.

**Supplementary information on the procedure of the spatial working memory task**

Testing took place in a quiet, dimly lit room. The SWM task was presented on a touch screen (ASUS T300F-FE006H, 12.5", 1366 × 768 pixels), which was placed on a desk in front of the participant. The set-up at both assessment sides, Berlin and Bonn, was identical. Standardized instructions were given orally by a trained experimenter, who explained that the goal of the task was to find a set of blue tokens hidden in a number of boxes and to use them to fill up an empty column on the right-hand side of the screen. Participants were also told that once a token was found, the next token was hidden in a different box. Importantly, the particular box in that the token has been found would not be used to hide another token, so that the participant had to avoid returning to any box in which a token has already been found. In a test problem set comprising three boxes, the experimenter ensured that the participant had understood the instructions correctly. All participants performed the task using the index finger of their dominant hand.

As SWM deficits appear to be more pronounced in demanding and complex SWM tasks, we employed a moderately difficult version of the CANTAB SWM test. The task comprised 11 problems presented in ascending order of difficulty (three 3-box problems, two 4-box problems, two 6-box problems, two 8-box problems, and two 10-box problems). In accordance with findings from previous research, only between-search errors and strategy scores were used as outcome measures. Between-search errors were recorded when a participant returned to a box in which a token has already been found. The strategy score was calculated by summing up the number of search sequences that start from a novel box for 6-, 8- and 10-box problems, indicating the inability to adopt a systematic searching approach. The most efficient method of completing the task is to start each search sequence from the same box and to search systematically through the boxes, returning to the initial box each time a token is found and excluding boxes from the search sequence.

**Supplementary Figures:**

**Figure S1:** The Spatial working memory (SWM) task (example: 8-box problem)


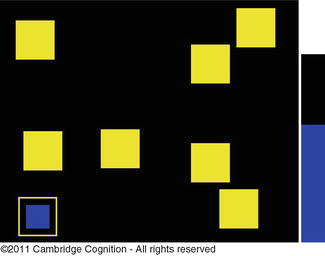


**Figure S2:** **Spatial working memory (SWM) performance.** Please note that higher values indicate worse SWM performance. **Panel A**: Estimated marginal means and standard errors from the ANCOVA model with the covariate age in the whole sample (N = 252), **panel B**: Estimated marginal means and standard errors from the ANOVA model in the age-matched subsample (N = 102). HC = healthy controls, REL = non-affected first-degree relatives of patients with OCD, OCD = patients with obsessive-compulsive disorder**.**  ** = p < .01

**Panel A**:

**
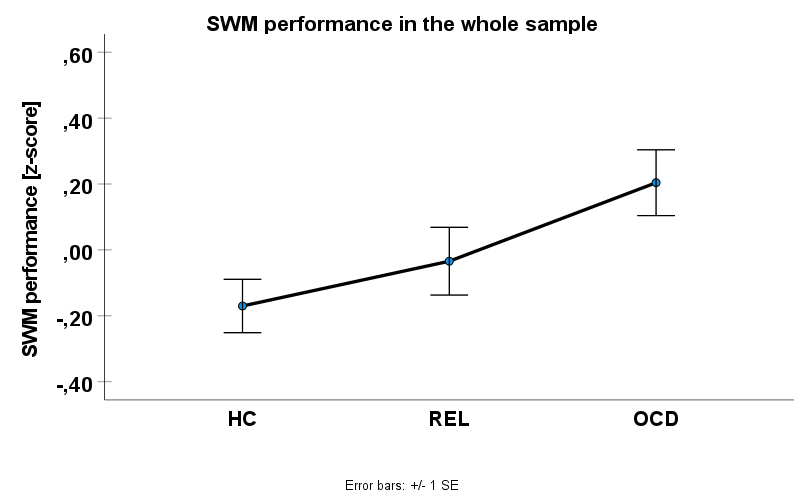
**

**

**Panel B**:


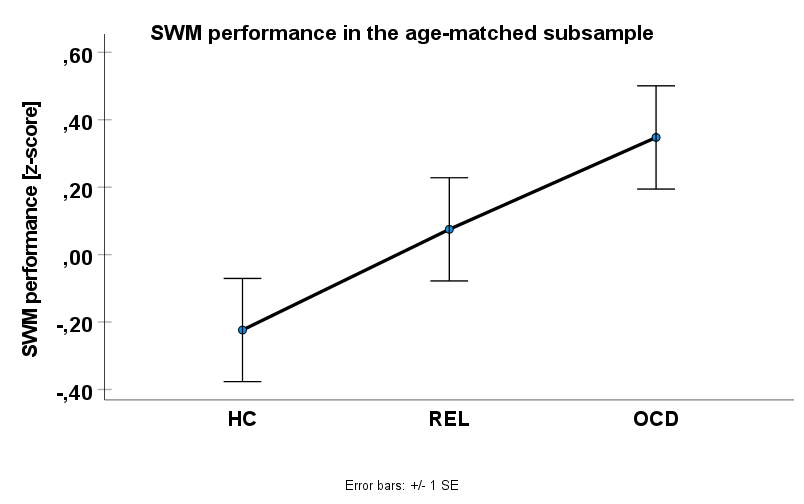


**

**Supplementary Tables**

**Table S1: Means and standard deviations of raw sum scores for strategy scores and between-search errors and raw z-scores for the combined measure of SMW performance.** HC = healthy controls, REL = non-affected first-degree relatives of patients with OCD, OCD = patients with obsessive-compulsive disorder.

|  | **Panel A:**  **Whole sample** [M(SD)] | | | **Panel B:**  **Age-matched subsample** [M(SD)] | | |
| --- | --- | --- | --- | --- | --- | --- |
| **Measure** | **HC** (N = 106) | **REL**  (N= 77) | **OCD**  (N= 69) | **HC**  (N = 34) | **REL**  (N= 34) | **OCD** (N= 34) |
| SWM strategy score | 30.10 (10.33) | 34.29 (10.77) | 34.97 (10.26) | 29.41 (11.07) | 33.15 (11.69) | 36.32 (9.94) |
| SWM between-search errors | 15.25 (10.52) | 22.81 (14.37) | 18.68 (12.08) | 17.41 (10.46) | 20.56 (13.64) | 23.71 (12.48) |
| SWM performance | -.28 (.79) | .22 (.95) | .09 (.86) | -.22 (.81) | .08 (.97) | .35 (.89) |

**Table S2: Supplementary results for the individual variables strategy score and between-search errors.** In the whole sample, results from the ANCOVA models including the covariate age are reported. In the age-matched subsample, results from the ANOVA models are reported. * significant at p < .05; ** significant at p < .01

| **Measure** | **Whole sample (N = 252)** | **Age-matched subsample (N = 102)** |
| --- | --- | --- |
| **Strategy scores:** | **Group effect:**  F(2, 248) = 5.07 p = .007, partial η² = .039  **Post-hoc comparisons:**  HC < REL: p = .472, d = .11  HC < OCD: p = .003, d = .47**  REL < OCD: p = .038, d = .35*  **Estimated marginal means  [mean (std. error), % difference from HC]:**  HC: 31.00 (1.02)  REL: 32.17 (1.29), + 3.8%  OCD: 35.96 (1.25), + 16.0% | **Group effect:**  F(2, 99) = 3.41, p = .037, partial η² = .064  **Post-hoc comparisons:**  HC < REL: p = .181, d = .33  HC < OCD: p = .009, d = .66**  REL < OCD: p = .232, d = .29 |
| **Between-search-errors:** | **Group effect:**  F(2, 248) = 2.16, p = .118, partial η² = .017  **Post-hoc comparisons:**  HC < REL: p = .259, d = .17  HC < OCD: p = .048, d = .31*  REL < OCD: p = .446, d = .13  **Estimated marginal means [mean (std. error), % difference from HC]:**  HC: 16.88 (1.14)  REL: 18.94 (1.45), + 12.2%  OCD: 20.49 (1.41), + 21.4% | **Group effect:**  F(2, 99) = 2.24, p = .112, partial η² = .043  **Post-hoc comparisons:**  HC < REL: p = .290, d = .26  HC < OCD: p = .028, d = .55*  REL < OCD: p = .324, d = .24 |

**Supplementary Results on correlations between SWM performance and obsessive-compulsive / depressive symptoms**

In the whole sample, worse SWM performance was related to higher obsessive-compulsive symptoms as assessed by the sum score of the OCI-R scale (r = .13, p = .041) and to higher depressive symptoms as assessed by the sum score of the BDI-2 (r = .14, p = .026). To explore the effect of OCD symptom dimensions, we tested the relationships between SWM performance and the six OCI-R subscales. We found that worse SWM performance was specifically associated with higher symptoms in the subscales ordering (r = .16, p = .010), and neutralizing (r = .17, p = .009). Symptoms of the other subscales were not significantly related to SWM performance (washing: p = .058; checking: p = .288; obsessing: p = .519; hoarding: p = .247).

In the age-matched subsample, worse SWM performance was also related to higher obsessive-compulsive symptoms (OCI-R sum score: r = .24, p = .016) but not to higher depressive symptoms (BDI-2 sum score: r = .13, p = .188). Higher symptoms in the OCI-R subscales washing (r = .24, p = .015), ordering (r = .26, p = .010), and neutralizing (r = .30, p = .002) were associated with worse SWM performance. The subscales checking (p = .104), obsessing (p = .349), and hoarding (p = .434) were not significantly related to SWM performance.

**Supplementary Results: ANCOVA with age and OCI-R sum score as covariates**

An additional ANCOVA in the whole sample including both age and OCI-R sum score as covariates and SWM performance as dependent variable, showed a non-significant group effect (F(2, 247) = .32, p = .723, partial η² = .003). Similarly, in the age-matched subsample, the ANCOVA with the covariate OCI-R sum score showed no significant main effect of group in SWM performance (F(2, 98) = .93, p = .398, η² = .019). When interpreting these results, it must be considered that group status (at least for OCD and HC) was mainly defined by the presence or absence of OCD symptoms. Thus, variance explained by group status is therefore largely eliminated when controlling for OCD symptoms.”

**Supplementary references:**

Structured Clinical Interview for DSM-IV TR (SCID):
First MB, Spitzer RL, Gibbon M, Williams JBW. *Structured clinical interview for DSM-IV axis I disorders-patient edition.* Biometrics Research Department, New York State Psychiatric Institute, 1995.

Family History Screen:
Weissman MM, Wickramaratne P, Adams P, Wolk S, Verdeli H, Olfson M. Brief Screening for Family Psychiatric History: The Family History Screen. *Arch Gen Psychiatry* 2000; **57**: 675–82.

Yale-Brown Obsessive Compulsive Scale (Y-BOCS):
Goodman WK, Price LH, Rasmussen SA, Mazure C, Fleischmann RL, Hill CL, *et al.* The Yale-Brown Obsessive Compulsive Scale. I. Development, use, and reliability. *Archives of General Psychiatry* 1989; **46**: 1006–11.

Obsessive-Compulsive Inventory-Revised (OCI-R):
Foa EB, Huppert JD, Leiberg S, Langner R, Kichic R, Hajcak G, *et al.* The obsessive-compulsive inventory: Development and validation of a short version. *Psychological Assessment* 2002; **14**: 485–96.

Beck Depression Inventory-II (BDI-II):
Beck AT, Steer RA, Brown GK. Beck Depression Inventory-ii (bdi-ii). *San Antonio, TX: Psychological Corporation* 1996.

Cambridge Neuropsychological Test Automated Battery (CANTAB):
Lowe, C., & Rabbitt, P. (1998). Test/re-test reliability of the CANTAB and ISPOCD neuropsychological batteries: theoretical and practical issues. *Neuropsychologia, 36*(9), 915-923.

Verbal IQ: WST: Schmidt, K.-H., & Metzler, P. (1992). *Wortschatztest (WST)*. Beltz.
